# Immune Characters and Plasticity of the Sentinel Lymph Node in Colorectal Cancer Patients

**DOI:** 10.1155/2021/5516399

**Published:** 2021-08-18

**Authors:** Xiaoyun Li, Jingling Tang, Hang Du, Xinjun Wang, Liyun Wu, Pingsheng Hu, Hua Zhang, Ruyi Zhang, Yuan Yang

**Affiliations:** ^1^Department of Anorectal Surgery, Affiliated Hospital of Guizhou Medical University, Guiyang 550004, China; ^2^Clinical Medical Research Center, Affiliated Hospital of Guizhou Medical University, Guiyang 550004, China; ^3^Department of Research and Development, Sinorda Biotechnology Co., Ltd., Guizhou 550004, China

## Abstract

**Purpose:**

This study is aimed at immunologically characterizing sentinel lymph nodes (SNs) in colorectal cancer (CRC) patients and identifying changes in immunological phenotype and function of SNs isolated from the tumor immunosuppressive microenvironment.

**Methods:**

A total of 53 pairs of matched SNs and non-SNs (NSNs) were collected by using a lymph node tracer dye. Flow cytometry was performed to detect the immunophenotype of T cells as well as the expression of activation and inhibitory markers. Differential expression and distribution of characteristic immune cell markers were analyzed by multiplex immunohistochemistry (mIHC). Transcriptomics analysis was conducted to compare the differences in the expression of immune-related genes among lymph nodes. The *ex vivo* culture of lymph nodes was carried out to examine changes in immunological phenotypes and functions.

**Results:**

Compared with NSNs, SNs harbored a significantly higher percentage of regulatory T cells (Tregs) but a lower proportion of MoMDSCs. As indicated in the mIHC assays, Tregs, T follicular helper (Tfh) cells, and M2 macrophages were mainly distributed in cortical areas, germinal centers, and subcapsular sinus areas, respectively, while significantly higher numbers of Tregs and Tfh cells were detected in SNs as compared to NSNs. Moreover, GSEA revealed that T cell activation genes and CD8+ T cell exhaustion-related genes are enriched in SNs and NSNs, respectively. The *ex vivo* culture led to an increase in the proportion of CD4+ cells, while activating T cells in SNs. In addition, SNs displayed a higher increase in the expression of cytokines IFN-*γ*, TNF-*α*, and sFas than NSNs.

**Conclusion:**

SNs are shown to be in an immune active state *in vivo*, while highly expressing inhibitory cytokines and suppressive markers. The *ex vivo* culture enhanced antitumor immunological function of SN-T cells, providing a starting material for adoptive cell therapy for CRC.

## 1. Introduction

Colorectal cancer (CRC) is the third most common diagnosed cancer and the fourth most deadly malignancy, with almost 900,000 deaths annually worldwide [1, 2]. As a heterogeneous disease of the intestinal epithelium, CRC involves multiple factors and stages, which are characterized by the accumulation of mutations and immune response disorders [3]. The pathogenesis of CRC is not fully understood. While the clinical treatment of CRC has been dominated by surgical resection, chemoradiotherapy, and targeted therapy, these methods often fail to eradicate tumor foci, resulting in tumor recurrence, progression, and poor prognosis. Therefore, alternative strategies need to be developed for the treatment of CRC patients.

Advancements in understanding of the complex interactions between tumor cells and the immune system have led to brand new approaches of immunotherapy. The body's immune system plays a role in immune surveillance, while it promotes the immune escape of tumors in certain stages, affecting tumor occurrence and development. After initial success in the treatment of melanoma, immunotherapy has been demonstrated as the most promising treatment option for various types of solid tumors, including CRC [4]. Immunotherapeutic drugs, especially immune checkpoint blockers related to T cell immunity, have been approved for clinical practice in various malignancies such as melanoma, non-small cell lung cancer, and many other epithelial tumors [5, 6]. Recent studies have shown that microsatellite instability-high CRC has a high response rate to immunotherapy [7, 8], suggesting a great potential of immunotherapy in CRC treatment.

Adoptive cell therapy (ACT) has several advantages over other types of cancer immunotherapy, which are dependent on the activation and amplification of a sufficient number of antitumor T cells in vitro and subsequent reinfusion of the cells to restore the antitumor activity of patients [9]. Lymphocytes used for ACT therapy are mainly derived from autologous tumor cells, such as tumor infiltrating lymphocytes (TILs). Conventional ACT therapy has certain drawbacks such as latent immune memory deficiency. In this case, ACT therapy for CRC faces the greatest challenge in obtaining TILs due to a number of reasons, including easy contamination of growing intestinal cancer tissues by bacteria because of being exposed to the intestinal lumen, a low success rate of TIL cultivation, and the presence of severe immunosuppression for TILs in tumors [10].

Sentinel node (SN) is considered as the primary station of tumor drainage. In SN, lymphocytes receive the earliest and a large amount of tumor antigen stimulation, generating a strong specific immune memory for tumor recognition. Thus, lymphocytes derived from SNs may become a good starting material for ACT, suggesting the great prospects and clinical potential of SN-T lymphocyte immunotherapy in the treatment of CRC [11, 12]. We previously showed that adjuvant SN-T lymphocyte immunotherapy is safe and feasible for postoperative CRC patients, while improving long-term survival of metastatic CRC [13]. Given that the scenario of using SN-T cells as good initiating cells for ACT lacks specific pairwise comparison data, this study was aimed at providing data support for SN-T cell-based ACT.

While the assessment of the immune status of cancer patients is mainly based on the analysis of the primary tumor and blood samples, data on immune characteristics of SNs are still lacking. In this study, we employed a number of biological methods and bioinformatics techniques to investigate specific immunological characteristics of SNs in CRC patients and to determine immunological phenotypes and function changes of SNs isolated from the tumor immunosuppressive microenvironment.

## 2. Materials and Methods

### 2.1. Patients

A total of 53 CRC patients diagnosed by colonoscopy or imaging were recruited from the Department of Anorectal Surgery at the Affiliated Hospital of Guizhou Medical University from October 2018 to July 2020. These patients did not receive neoadjuvant therapy (radiotherapy or chemotherapy) prior to radical resection of CRC. The study protocol was reviewed and approved by the hospital ethical committee. All patients provided written informed consent to participate in the study.

### 2.2. Lymph Node Collection

For each patient, 1 ml of patent blue V tracer was injected into the subserosa at four points surrounding the tumor within 5 min after tumor tissue isolation. Blue-stained lymph nodes within the first 3 min following dye application were marked with a suture and collected *ex vivo* as SNs, while lymph nodes showing no blue staining near the tumor were referred to as nonsentinel nodes (NSN). The SN and NSN were studied in pairs in each patient. The lymph nodes were excised and cut in half; one half was retained for analysis, and the remaining half was processed for routine histopathological examination. Routine HE staining and immunohistochemistry or flow cytometry was performed to detect the expression of Epcam and CK in all paired lymph nodes [14], except 7 pairs of metastatic lymph nodes (Table 1). A portion of each tumor tissue was used for antigen preparation [15].

### 2.3. Flow Cytometry Analysis

Single cell suspensions for 13 pairs of lymph nodes were obtained by gentle pressure using a loose fit glass homogenizer. The following fluorescent-labeled monoclonal antibodies (mAbs) were used for analysis of immune cell subpopulations and T cell function: CD3, CD4, CD8, CD19, CD25, CD127, FoxP3, Helios, CD45RA, HLA-DR, CD15, CD33, CD11b, CD14, OX40, ICOS, CD28, CD137, GITR, PD1, CTLA4, TIGIT, and LAG3 (BioLegend) (Table S1). For surface markers, cells were incubated with mAbs for 30 min at 4°C according to the manufacturer's instructions. For intracellular markers, cells were fixed, permeabilized, and blocked using a Cytofix/Cytoperm Solution Kit (BD Pharmingen) prior to staining with mAbs. Dead cells were excluded by using the Zombie Yellow™ Fixable Viability Kit (BioLegend). After incubation, cell suspensions were washed with phosphate-buffered saline (PBS), and then, the cell pellets were resuspended in 0.5 ml PBS for analysis. Thereafter, samples were further analyzed using a Navios flow cytometer (Beckman Coulter) and FlowJo software (Figure S1).

### 2.4. Multiplex Immunohistochemistry

Six pairs of lymph nodes were randomly selected, formalin-fixed, paraffin-embedded, and subjected to multiplex immunohistochemistry (mIHC). The mIHC was performed by using a PANO 7-plex IHC kit (Panovue, China) following the standard protocol [16]. Immune cell panels included the following antibodies: CD3 (1 : 200, Abcam, ab16669), CD8A (1 : 300, Cell Signaling Technology, 70306), Foxp3 (1 : 500, Abcam, 20034), PD1 (1 : 50, Cell Signaling Technology, 43248), and CD163 (1 : 100, Cell Signaling Technology, 93498). The slides were incubated with the primary antibodies, followed by 0.3% hydrogen peroxide solution for blocking endogenous peroxidase. DAPI (Sigma-Aldrich) was used for nuclear counterstaining. Images were acquired and analyzed by using a Mantra System (PerkinElmer) and inForm image analysis software (PerkinElmer), respectively.

### 2.5. Difference Analysis of Expression Spectrum

Part of SNs and NSNs were maintained in an RNA storage solution at room temperature overnight and then transferred to -80°C. A total of 27 pairs of lymph nodes were randomly selected. RNA extraction, library construction, and sequencing were carried out as described previously [17]. Briefly, 20 mg of total RNA was extracted, and cDNA libraries were prepared using NEBNext® Ultra™ RNA Library Prep Kit for Illumina® (NEB, USA). The libraries were sequenced on an Illumina platform, and 150 bp paired-end reads were generated.

Gene set enrichment analysis (GSEA) software was used to perform functional analyses. Gene set analysis was conducted by the clusterProfiler R package (v3.14.3) based on C7 (immunologic gene sets) from the Molecular Signatures Database (MSigDB v7.1.1). GSEA was first applied to the ranking that was defined by the log2 fold change (log2FC) of the differential expression analysis using DESeq2. The entire ranked list was used to calculate an enrichment score for each gene set, which reflects how the genes in each set are distributed in the ranked list. Normalized enriched score (NES) was determined for each gene set. The significant enrichment of gene set was identified based on the absolute values of NES > 1, *p* ≤ 0.05, and *p* adjust ≤ 0.25.

### 2.6. Cytokine Analysis

*Ex vivo* cell culture was performed as described previously [13]. The ELISpot assay for IFN-*γ* was carried out using precoated ELISpot kits (Mabtech) and a standardized detection system (Autoimmun Diagnostika GmbH) according to the manufacturer's instructions. Briefly, 50,000 cultured cells were plated overnight on a 96-well plate precoated with anti-IFN-*γ* antibody in IL-2-containing medium. On day 9 after culture, the cells were plated and stimulated with tumor autoantigen for 48 h. The production of IFN-*γ*, TNF-*α*, soluble Fas (sFas), IL-2, granzyme A, granzyme B, granulysin, and perforin was determined by using the LEGENDplex™ Multi-Analyte Flow Assay Kit (BioLegend) as described previously [18].

### 2.7. Statistical Analyses

The data were statistically analyzed using GraphPad Prism 8.0.2 software. Categorical variables were tested by the chi-square test, while quantitative data were analyzed by paired *t*-test and presented as mean ± SD. The difference was considered significant at *p* < 0.05.

## 3. Results

### 3.1. Immunological Features of Lymph Nodes

**Figure 1:**
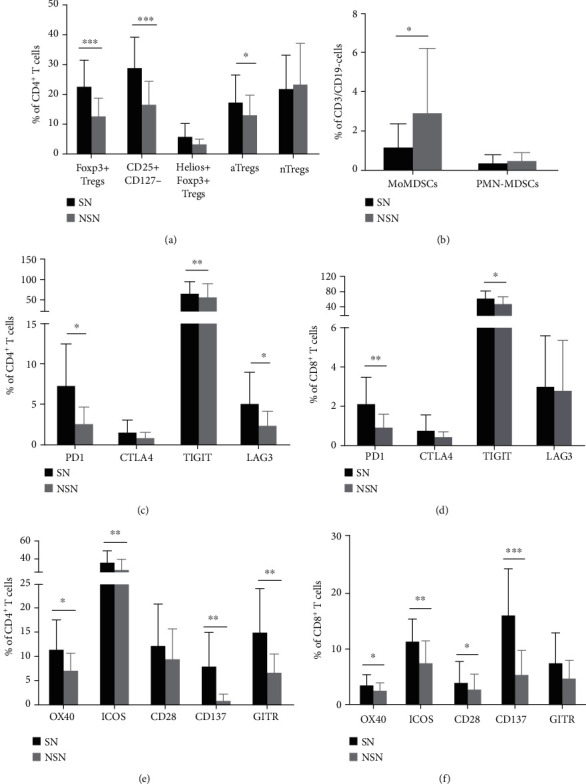
Immunological features of lymph nodes. The proportions of the subsets of Tregs (Foxp3+ Tregs, CD25+CD127-, Helios+Foxp3+ Tregs, aTregs, and nTregs) (a) and MDSC (MoMDSCs and polymorphonuclear MDSCs, PMN-MDSCs) (b) in SN and NSN are shown. The expression of CD4+ and CD8+ T cell inhibitory markers (c, d) and activation markers (e, f) in SN and NSN is depicted. ^∗^*p* < 0.05, ^∗∗^*p* < 0.01, ^∗∗∗^*p* < 0.001.

**Figure 2:**
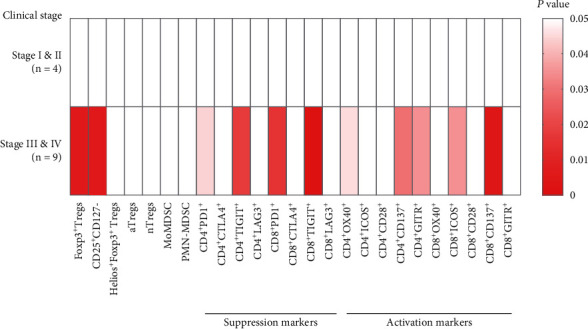
Immunological features of SN and NSN in patients at different clinical stages.

The subtypes of immunocytes present in the SN and corresponding NSN were determined by flow cytometry. Regulatory T cells (Tregs) are highly heterogenous and can be further classified into five subsets: Foxp3+ Tregs, CD25+CD127-, Helios+Foxp3+ Tregs, activated Tregs (aTregs, Foxp3+CD45RA-), and naïve Tregs (nTregs, Foxp3intCD45RA+) [19]. As depicted in Figures 1(a) and 1(b), there was a significant difference in immunocyte composition between the SN and matched NSN. Notably, compared with the NSN, SN harbored markedly higher percentages of Foxp3+ Tregs, CD25+CD127-, and aTregs subsets, but a significantly lower percentage of monocytic MDSCs (MoMDSCs, CD3-CD19-CD14+HLA-DRlow/-). To evaluate the function of T cells in SN and NSN, we further analyzed the expression of indicators for T cell activation (OX40, ICOS, CD28, CD137, and GITR) and markers for the inactivation (PD1, CTLA4, TIGIT, and LAG3). As shown in Figures 1(c)–1(f), a significant increase in the expression of three immunosuppressive markers PD1, TIGIT, and LAG3, as well as five activation markers OX40, ICOS, CD137, CD28, and GITR, was detected in SN as compared to the NSN. According to the influence of lymph node metastasis on clinical stages, all patients were divided into two groups: stages I and II (no lymph node metastasis) and stages III and IV (lymph node metastasis). We further compared immunological characteristics between SN and NSN in each group. As presented in Figure 2, in the stage III and IV patients, SN displayed significantly higher levels of Tregs, T cell inhibitory markers, and activation markers than the matched NSN. Meanwhile, there were no differences in immunological characteristics between SN and NSN in the group of stages I and II.

Next, a follow-up study was conducted in the 13 patients. As illustrated in Table S2, we failed to perform statistical analysis on the differences in prognosis and immunological characteristics between the two groups of patients because of the short-term follow-up (approximately 1 year). However, we observed that among the patients, one case with progressive disease (death) exhibited very different immunological characteristics of the lymph node compared with the others, as indicated by the differences in the expression levels of T cell activation and inhibition markers between the SN and matched NSN.

**Figure 3:**
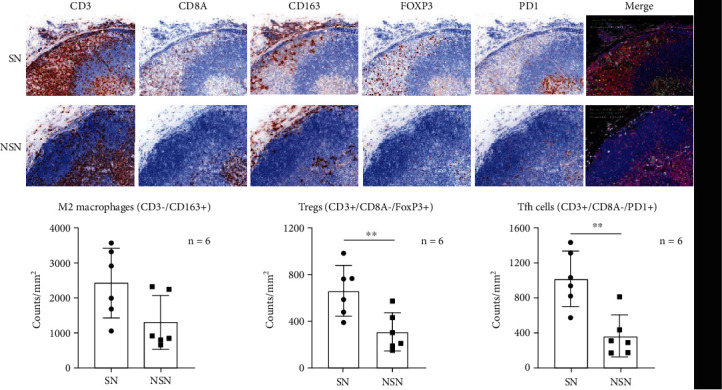
Spatial distribution and correlations of characteristic immune cell markers in lymph nodes. The expression of M2 macrophages (CD3-/CD163+), Tregs (CD3+/CD8A-/FoxP3+), and Tfh cells (CD3+/CD8A-/PD1+) is shown in the histogram. ^∗∗^*p* < 0.01.

**Figure 4:**
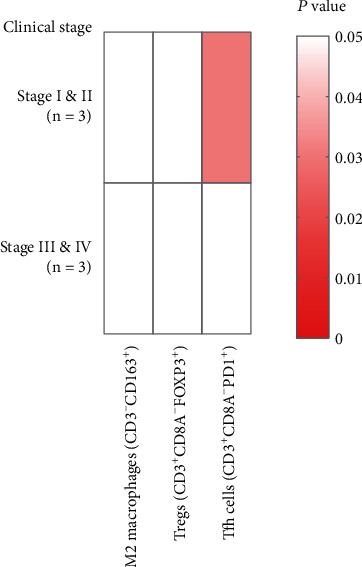
The expression of M2 macrophages (CD3-/CD163+), Tregs (CD3+/CD8A-/FoxP3+), and Tfh cells (CD3+/CD8A-/PD1+) in SN and NSN of patients at different clinical stages.

To analyze spatial distribution and correlations of characteristic immune cell markers in lymph nodes, the expression of CD3, CD8A, Foxp3, PD1, and CD163 was examined by mIHC. As shown in Figure 3, Tregs (CD3+/CD8-/FoxP3+) and T follicular helper (Tfh) cells (CD3+/CD8-/PD1+) in lymph nodes were mainly distributed in cortical areas and germinal centers, respectively. M2 macrophages (CD3-/CD163+) in lymph nodes were distributed in the subcapsular sinus and cortical areas, in which M2 macrophages in SN were more concentrated in subcapsular sinus areas. Clearly, SN harbored significantly higher numbers of Tregs and Tfh cells than NSN, while there was no significant difference in the expression of M2 macrophages between SN and NSN. Moreover, we showed that among stage I and II patients, the number of Tfh cells was significantly increased in SN as compared to NSN, while it was not the case in patients at stages III and IV. Notably, there was no difference in the number of M2 macrophages and Tregs between SN and NSN in the two groups (Figure 4).

### 3.2. Gene Set Enrichment Analysis

**Figure 5:**
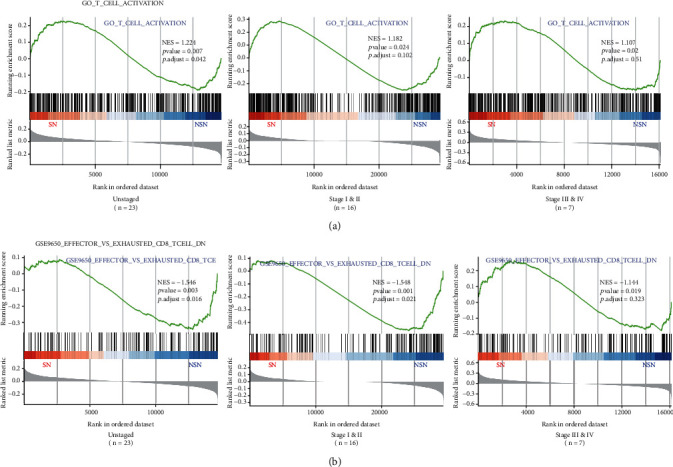
GSEA enrichment analysis of signatures of T cell activation genes (a) and CD8+ T cell exhaustion-related genes (b).

After excluding 4 pairs of outlier samples, GSEA analysis was performed on 23 pairs of samples to identify the differences in the enrichment expression of lymphocyte activation and inhibition genes between SN and NSN. As depicted in Figure 5, signatures of T cell activation and CD8+ T cell exhaustion were differentially enriched in SN and NSN, respectively. Furthermore, we observed that the enrichment trend of T cell function-related genes in the lymph nodes of patients at stages I and II remained unchanged following the staging of patients. Conversely, we failed to observe the above enrichments in patients at stages III and IV.

### 3.3. Profiles of Surface Markers in Ex Vivo-Cultured SN-T Cells

**Figure 6:**
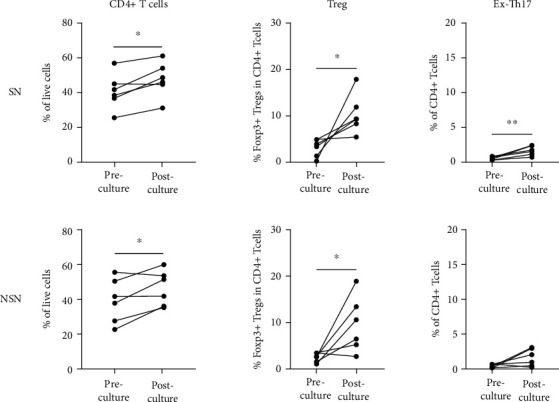
Changes in immunophenotype of lymph nodes after *ex vivo* culture. ^∗^*p* < 0.05 and ^∗∗^*p* < 0.01.

**Figure 7:**
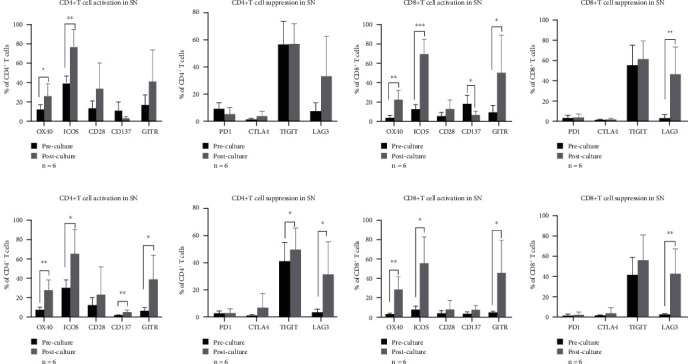
Changes in T cell function of SN and NSN caused by *ex vivo* culture. ^∗^*p* < 0.05, ^∗∗^*p* < 0.01, and ^∗∗∗^*p* < 0.001.

To investigate changes in immunological phenotypes and functions of lymph nodes after *ex vivo* culture, we evaluated the subsets and functional markers of cultured lymphocytes on the 9th day. As presented in Figure 6, *ex vivo* culture led to an increase in the proportion of CD4+ cells mainly involving Tregs and Th17 cells in SN, while it increased the proportion of CD4+ cells mainly involving Tregs in NSN. Significantly upregulated expression of OX40, ICOS, and GITR in CD4+ and CD8+ cells suggested the initiation of T lymphocyte activation in lymph nodes. However, the proportion of CD137 cells was significantly decreased in SN as compared to NSN, while there was almost no significant change in T cell inhibition in SN (Figure 7).

### 3.4. Changes in Cytokine Levels after Ex Vivo Culture

**Figure 8:**
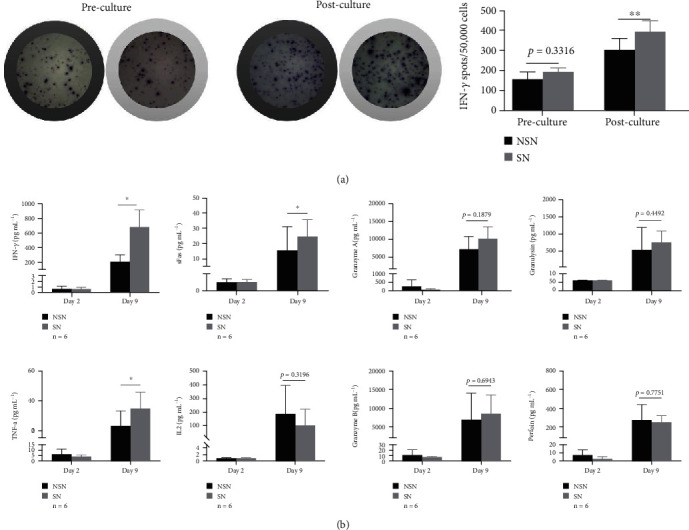
Cytokine changes induced by *ex vivo* culture. (a) ELISpot assay was performed to detect IFN-*γ* secretion stimulated by autologous tumor antigen. (b) Production of IFN-*γ*, IFN-*α*, sFas, IL-2, granzyme A, granzyme B, granulysin, and perforin on the second and ninth days of *ex vivo* culture was measured by using LEGENDplex. ^∗^*p* < 0.05.

To evaluate tumor-specific responses of lymphocytes to autologous tumor lysates, we firstly performed IFN-*γ* ELISpot assays. A comparison of IFN-*γ* expression between the starting culture and the ending culture revealed that stimulation with the autologous tumor antigen led to a high level of spontaneous IFN-*γ* secretion. Moreover, SN cells contained a significantly higher level of IFN-*γ* than NSN counterparts (Figure 8(a)). Next, we conducted the LEGENDplex assay to detect the expression of other selected cytokines. As shown in Figure 8(b), while the expression levels of cytokines on the 2^nd^ day of *ex vivo* culture remained relatively low, the cells secreted large amounts of cytokines on the 9th day of *ex vivo* culture. In this case, SN exhibited a significantly higher increase in the expression of lethal cytokines IFN-*γ*, TNF-*α*, and sFas than NSN.

## 4. Discussion

ACT therapy takes full advantage of highly specific targeting of T cells. In this case, progenitors of patient's own or allogeneic antitumor effector cells were firstly amplified in vitro for restoring their antitumor activity, and the activated T cells were then reinfused into patients for an antitumor therapy. This therapy has shown promising initial results in the treatment of various tumors [20, 21]. However, due to the disadvantages of TILs in CRC, the application of ACT therapy in CRC has been limited. In addition, several studies suggested that TILs are not the main antitumor lymphocytes [22, 23]. Given the properties of SNs, we reasoned that SNs may be the “headquarters” of antitumor immunity. In this study, TILs were replaced by SN-derived tumor reactive T cells (SN-T cells) due to the obvious advantages of SN over TILs [24]. And this study indicated that SN-T cells might be used as initiation materials for ACT therapy in CRC.

In-depth exploration on the relationship between SN and immune regulation has shown that the relationship may be closely related to tumor progression. A study on melanoma revealed that compared with the NSN, SN from the same nodal basin displayed a downregulated antitumor immunity, while it harbored a significantly increased number of immunosuppressive Foxp3+ Tregs as well as a decreased number of immunogenic CD11c+ conventional tree dendritic cells and CD86+ mature dendritic cells [25]. Another study on breast cancer identified a significant increase in the ratios of MDSCs, Tregs, and “exhausted” CD4+ and CD8+ T cells in SN, indicating that SN has a profound immunosuppressive microenvironment, which becomes more profound in the presence of metastases [26]. In the present study, we sought to map the immune landscape of SN in CRC. Similar to the findings in previous reports, we observed an increased proportion of Tregs as well as a significantly higher expression of FoxP3 in tumor SN as compared to the corresponding NSN. However, we found a decrease in the proportion of MoMDSCs in SN, which might promote T cell immunity through various metabolic effects [27]. Meanwhile, an increase in Tfh cells in SN could enhance antitumor immunity [28]. Here, we observed that in SN, M2 macrophages, a type of immunosuppressive cells, were clustered in subcapsular sinus areas of lymph nodes, the main functional area for antigen presentation [29]. This observation suggested that SN harbors an immunosuppressive microenvironment, and the M2 aggregation may be a response to tumor antigen stimulation. In addition, we found that compared with NSN, SN highly expressed three immunosuppressive markers, PD1, TIGIT, and LAG3, as well as five immune activation markers, OX40, ICOS, CD137, CD28, and GITR. Therefore, while SN is immunologically active *in vivo*, it highly expresses inhibitory cytokines and activating markers because of its immunosuppressive microenvironment. Subsequently, we further compared the immunological characteristics between SN and NSN in stage I and II (no lymph node metastasis) and stage III and IV groups, respectively. Interestingly, the similar characteristics as above were observed in SN and NSN in stage III and IV patients, while there were no differences in immunological characteristics between SN and NSN in stage I and II patients, which might be related to the small sample size. The above findings in the present study were justified by the transcriptomic analysis showing that T cell activation genes were enriched in SN. Besides, CD8+ T cell exhaustion-related genes were found to be enriched in NSN, implying that CD8+ T cells may play a major antitumor effect. These enrichments were also identified in patients at stages I and II but not observed in patients at stages III and IV.

In this study, we stimulated the *ex vivo* cultured lymph nodes and analyzed changes in their immunological phenotypes and function in order to verify the antitumor immune activity of SN. The study showed that the in vitro culture of SN led to an increase in the proportion of CD4+ cells mainly involving Tregs and Th17 cells. Given that Foxp3 is increased after *ex vivo* stimulation, but not very inhibitory [30, 31], we did not equate increased Foxp3 with the increased inhibition of Tregs. Moreover, we found an increased expression of OX40, ICOS, and GITR, suggestive of promoted T cell activation. van Pul et al. reported that in vitro immunomodulation of Toll-like receptor-9 agonist CpG-B on SLN can effectively overcome immunosuppression by preferentially activating lymph node-resident dendritic cells to restore antitumor immunity, thereby ensuring breast cancer-specific T cell response [32]. In the present study, we observed that after *ex vivo* culture, SN displayed a higher increase in the expression of IFN-*γ*, TNF-*α*, and sFas than NSN. Among them, IFN-*γ* can coordinate T cell responses through its unique effects on distinct non-T cell target cells. For example, IFN-*γ* is capable of controlling T cell expansion via signaling in dendritic cells [33]. TNF-*α* induces tumor cell apoptosis by binding to tumor necrosis factor-related ligands, while binding of Fas ligands on cytotoxic T lymphocytes with their receptors elicits apoptosis [34]. Collectively, these data suggest that the *ex vivo* culture of SN isolated from the original environment could enhance the antitumor immunological function of T cells.

## 5. Conclusions

In short, we investigate immunological characteristics of SN in CRC patients and find that SN is in a state of immune activation in the body. Removal of SN from the tumor immunosuppressive microenvironment leads to an enhancement of its immunological function. These findings highlight the uniqueness and specificity of SN in facilitating cancer immunotherapy and developing a promising SLN-T cell-based immunotherapy for CRC.

## Figures and Tables

**Table 1:** Demographic and pathological characteristics of CRC patients (*N* = 46).

Characteristic	*N*	%
Gender		
Male	25	54.35
Female	21	45.65
Age (median, range)	64.04(34-90)	
Tumor size (cm; median, range)	4.77(0.2-11)	
Median preop CEA (range)	10.81(0.31-74.25)	
Tumor location		
Cecum colon	6	13.04
Ascending colon	13	28.26
Transverse colon	3	6.52
Descending colon	3	6.52
Sigmoid colon	8	17.39
Rectum	13	28.26
Tumor (T) stage		
T2	5	10.87
T3	40	86.96
T4b	1	2.17
N stage		
N0	26	56.52
N+	20	43.48
M stage		
M0	42	91.30
M1	4	8.70
Clinical stage		
I	5	10.87
II	21	45.65
III	16	34.78
IV	4	8.70
Microsatellite instability (MSI)		
MSI-H	9	19.57
MSI-L/MSS	37	80.43

MSI-H: high-frequency MSI; MSI-L: low-frequency MSI; MSS: microsatellite stable.

## Data Availability

The data used and/or analyzed during the current study are available from the corresponding author on reasonable request.

## Supplementary Materials

Supplementary Materials Table S1: flow cytometry antibody combination use table. Table S2: the follow-up information of CRC patients (*N* = 13). Figure S1: gating strategy from one representative SN cell.
