# Supplementary material for: Immune Characters and Plasticity of the Sentinel Lymph Node in Colorectal Cancer Patients
Source: J Immunol Res. 2021 Aug 18;2021:5516399. doi: 10.1155/2021/5516399 (PMC8390165; doi:10.1155/2021/5516399)
Supplement: Supplementary Materials — Table S1: flow cytometry antibody combination use table. Table S2: the follow-up information of CRC patients (N = 13). Figure S1: gating strategy from one representative SN cell. [file 5516399.f1.docx]

**Table S1. Flow cytometry antibody combination use table**

| **Fluorochrome** | **Tregs** | **MDSCs** | **T cell suppression** | **T cell activation** |
| --- | --- | --- | --- | --- |
| FITC | FoxP3^#^ | / | PD1 | OX40 |
| PE | Helios^#^ | HLA-DR | CTLA4^#^ | ICOS |
| ECD | CD45RA | / | CD3 | CD3 |
| PerCP-Cy5.5 / PC5 | CD4 | CD3/CD19 | CD4 | CD28 |
| PE-Cy7 | / | CD15 | / | / |
| APC | CD127 | CD33 | TIGIT | CD137 |
| APC-Cy7 | CD8 | CD11b | CD8 | CD8 |
| PacificBlue / BV421 | CD25 | CD14 | LAG-3 | GITR |
| Zombie Yellow | DCM | DCM | DCM | DCM |

^#^ Intracellular markers

**Table S2. The follow-up information of CRC patients (N=13)**

| **Patient ID.** | **Age/sex** | **AJCC stage/ TNM classification** | **Patient Enrolled** | **Patient Last Contact** | **Months Last Response** | **Response Last** |
| --- | --- | --- | --- | --- | --- | --- |
| JISJ | 66/F | ⅢB/T3N1bM0 | 2020-05-13 | 2021-05-14 | 12 | CR |
| ZHKA | 34/M | ⅡA/T3N0M0 | 2020-05-15 | 2021-05-14 | 12 | CR |
| XITW | 72/F | ⅢB/T3N1cM0 | 2020-05-20 | 2021-05-14 | 11.8 | CR |
| ZHKZ | 78/M | ⅡA/T3N0M0 | 2020-05-28 | 2021-05-14 | 11.6 | CR |
| XUGZ | 90/F | ⅢB/T3N1aM0 | 2020-06-01 | 2021-05-14 | 11.5 | CR |
| FAQK | 63/F | ⅡA/T3N0M0 | 2020-06-03 | 2021-05-14 | 11.3 | CR |
| WAHY | 70/F | ⅢB/T3N1bM0 | 2020-06-10 | 2021-05-14 | 11.1 | CR |
| YILF | 81/F | ⅡA/T3N0M0 | 2020-06-11 | 2021-05-14 | 11.1 | CR |
| TUGH | 63/F | ⅢB/T3N1aM0 | 2020-06-12 | 2021-05-14 | 11.1 | CR |
| CHGZ | 70/F | ⅢB/T3N1cM0 | 2020-06-17 | 2021-05-14 | 10.9 | CR |
| ZHQY | 72/M | ⅢB/T3N2aM0 | 2020-06-18 | 2021-05-14 | 10.9 | PD^#^ |
| HUCC | 55/M | ⅢB/T3N1aM0 | 2020-06-28 | NA | 10.5 | NA |
| WAXY | 61/F | ⅢB/T3N1aM0 | 2020-07-14 | 2021-05-14 | 10 | CR |

Complete response (CR), progressive  [disease](C:/Program%20Files%20(x86)/Youdao/Dict/8.9.6.0/resultui/html/index.html#/javascript:;) (PD), NA (not available)

^#^The patient died


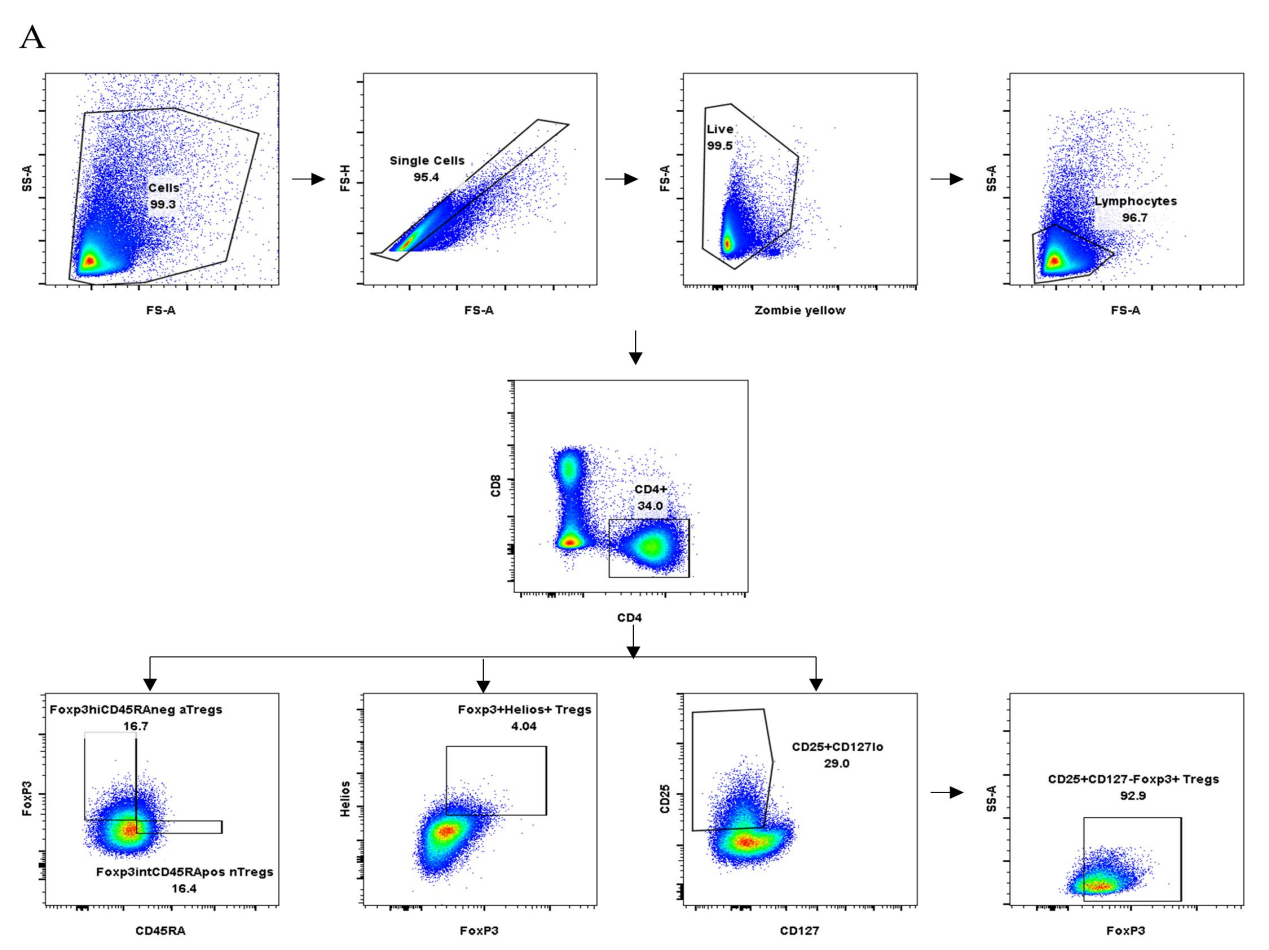


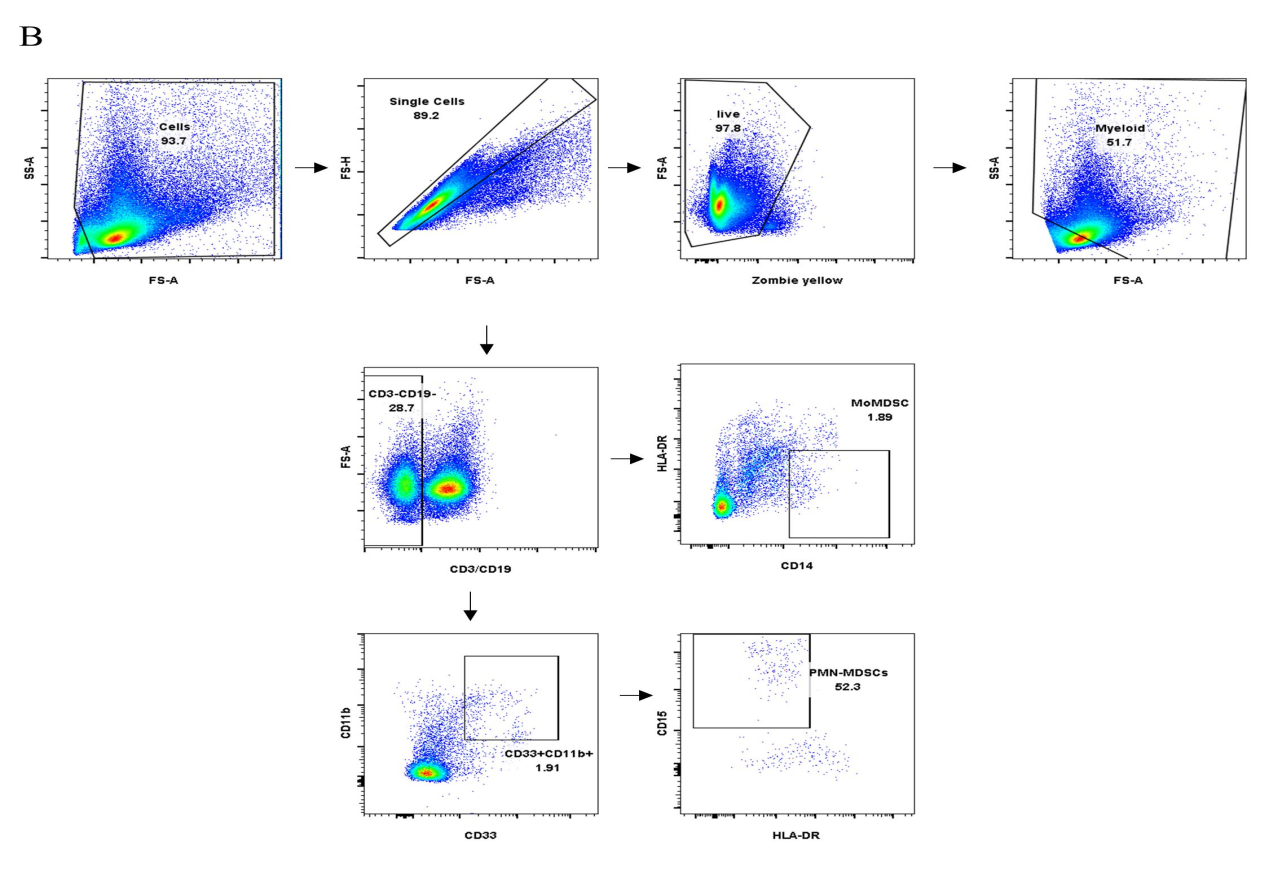

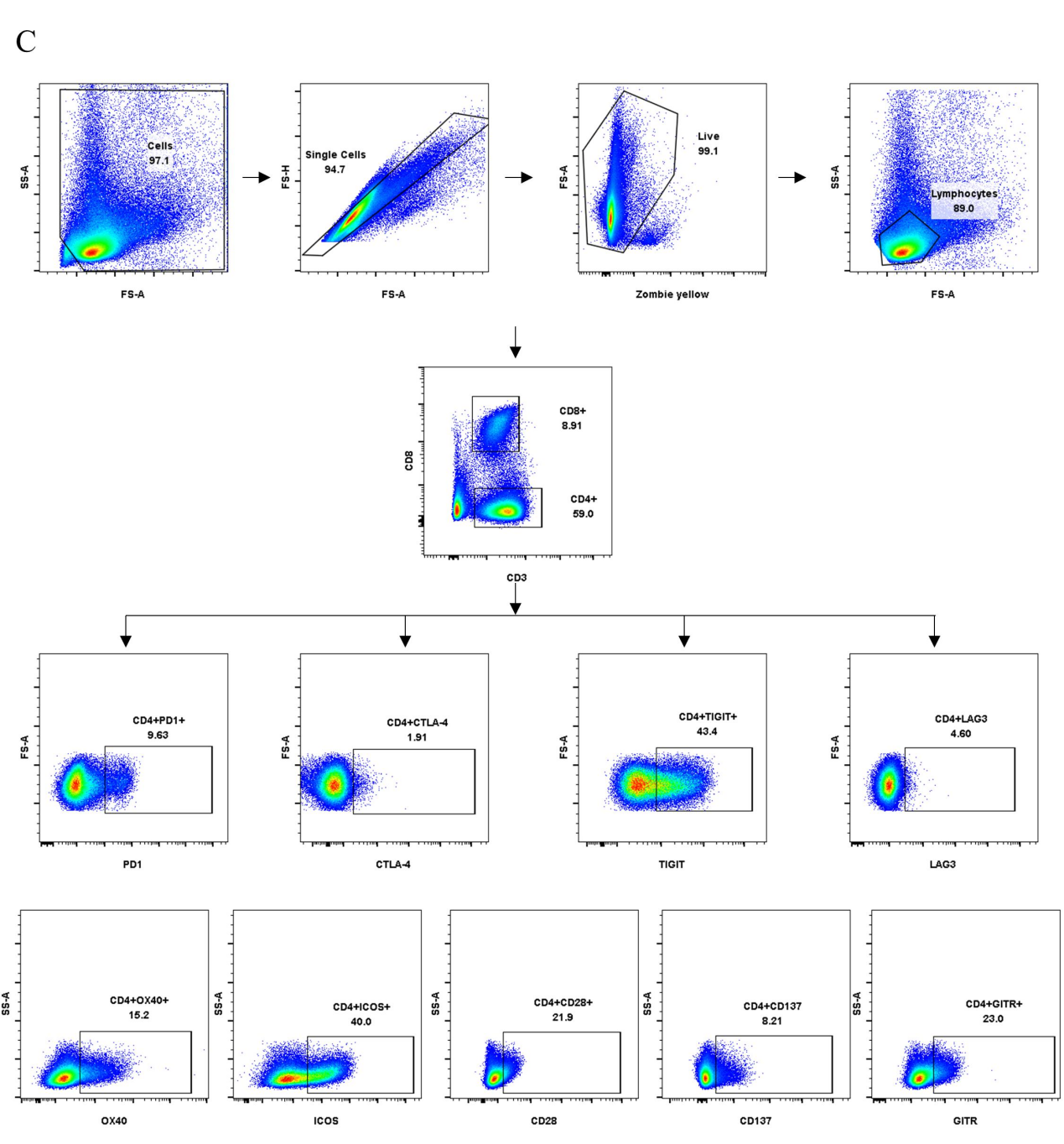


**Figure S1. Gating strategy from one representative SN cell**

Tregs subsets (**A**). MDSC subsets (**B**). T cell suppression markers and T cell activation markers (**C**). Fluorescence minus one (FMO) was used to determine the positive gate.
